# Evaluation of Low-Cost Smartphone-Based Infrared Cameras to Assess the Cooling and Refrigerated Storage Temperatures of Fresh Produce

**DOI:** 10.3390/foods11213440

**Published:** 2022-10-30

**Authors:** Boran Yang, Govindaraj Dev Kumar, Kevin Mis Solval

**Affiliations:** 1 Department of Food Science and Technology, University of Georgia, Griffin, GA 30223, USA; 2 Center for Food Safety, The University of Georgia, Griffin, GA 30223, USA

**Keywords:** thermal imaging, smartphone-based infrared cameras, fresh produce, simulated hydrocooling, temperature monitoring

## Abstract

Populations of pathogens may increase in fresh produce when subjected to temperature abuse. Smartphone-based infrared (SBIR) cameras are potential alternatives for temperature measurements of fresh produce during postharvest handling and storage. This study compared the performance of SBIR cameras (FLIR and Seek) against conventional temperature acquisition devices for evaluating fresh produce’s simulated hydrocooling and storage conditions. First, thermal images of fresh produce were obtained with SBIR cameras and handheld thermal imagers at ~35 °C, ~20 °C, and ~4 °C to simulate outdoor, packinghouse, and refrigerated environments, respectively. Next, fresh produce was incubated at ~42 °C for 20 h and immersed in chilled water for a hydrocooling simulation. Then, boxes containing cooled fresh produce were stored in a walk-in cooler at different heights for three days. FLIR SBIR cameras were more effective at capturing thermal images of fresh produce than Seek SBIR cameras in all evaluated conditions. More importantly, SBIR cameras accurately acquired temperature profiles of fresh produce during simulated hydrocooling and cold storage. Additionally, the accuracy and quality of thermal images obtained with FLIR cameras were better than those obtained with Seek cameras. The study demonstrated that SBIR cameras are practical, easy-to-use, and cost-effective devices to monitor fresh produce’s temperature during postharvest handling and storage.

## 1. Introduction

The consumption of fresh produce has increased since the 1980s due to modern consumers’ health and wellness awareness [1]. According to Doona et al. [2], fresh produce is a fast-growing food segment that appeals to the health-conscious consumer as nutritious, convenient, and tasty. Nevertheless, a higher demand for fresh produce brings new challenges to the industry because these products are minimally processed and often eaten raw, increasing the probability for foodborne pathogens to cause outbreaks that could affect consumers [3]. Therefore, the microbial safety of fresh fruits and vegetables is always a concern for the fresh produce industry [4]. Ready-to-eat (RTE) fresh produce have been increasingly implicated in outbreaks and are considered potential vectors for the transmission of pathogenic microorganisms [5]. According to the US Center for Disease Control and Prevention (CDC), around 10% of the total foodborne outbreaks in the US were associated with the consumption of raw vegetable crops (e.g., prepackaged leafy greens, salad mixes, and romaine lettuce) and fruits in 2016 [6]. Hence, controlling microbial growth in fresh produce during postharvest handling and storage is essential for ensuring quality and food safety.

Postharvest handling of fresh produce (necessary for extending shelf life; while minimizing product damage, quality deterioration, and microbial growth) includes, but is not limited to, rapid cooling, washing, disinfecting, slicing/cutting, packaging, and cold storage [7]. While raw produce can carry significant microbial loads upon harvest, microbial contamination can occur during postharvest processing through contaminated water, equipment, containers, knives, human hands, and gloves [1]. Not surprisingly, foodborne pathogens can survive and grow in postharvest handling, and low levels may even reach the final raw products. Therefore, rapid cooling is critical in the postharvest handling of fresh produce because it can minimize microbial growth and spoilage [1]. Often, fruits and vegetables are cooled after harvesting to reduce “field heat” and minimize quality deterioration caused by enzymatic activity and spoilage bacteria [8]. This process may also reduce foodborne pathogens’ growth (if present). The rapid cooling of fresh produce may be carried out with chilled water or ice. Often, the temperature of the coolant is the only parameter monitored during cooling, while the fresh produce temperature is ignored. Moreover, it is conventionally assumed that the coolant temperature is comparable to that of fresh produce; however, this may not always be the case and could result in improper/incomplete cooling. In storage, fresh produce generates heat primarily by respiration [9]. During this process, fruits and vegetables utilize their local reserves (e.g., carbohydrates) as a source of energy and use the oxygen from the air to keep them alive; this heat is known as “vital heat” [10]. According to Aggarwal, Mohite, and Sharma [11], some fruits and vegetables have higher respiration rates (e.g., spinach and asparagus) than others (e.g., grapes, apples, potatoes). While higher respiration rates may increase the temperature of fresh produce during refrigerated storage, effective temperature monitoring systems can help minimize fresh produce’s temperature abuse by helping to develop effective intervention strategies.

Temperature measurements in food processing facilities are acquired with conventional thermometers, thermocouples, single-point infrared thermometers, and resistance temperature devices. However, these single-point temperature instruments may require contacting the targeted object [12]. In contrast, thermal imaging (TI) or infrared thermography, a two-dimensional, noninvasive, and noncontact technique, has recently been introduced to quantify the changes in surface temperature of objects with a high spatial and temporal resolution in real-time [13,14,15]. Thermal images are produced with infrared (IR) cameras that collect electromagnetic radiation (wavelengths of 0.9–14 µm) emitted by the targeted objects/scenes [16]. Higher emitted radiation results in higher temperature values. Unlike other high-tech cameras that have recently been introduced into the market (e.g., hyperspectral), the performance of IR cameras is not affected by visible light and does not require unique light sources [17]. Hence, they are powerful devices that can be used to collect temperature variations across an object or scene under different environmental conditions. TI has been gaining popularity in the food industry due to the development of new thermal technologies that have significantly reduced the cost of IR cameras and simplified their operation [13]. Even more, the development of high-tech smartphones, the availability of modern communication networks and the introduction of low-cost smartphone-based infrared (SBIR) cameras into the market have created a whole new realm of possibilities and opportunities to develop applications of these technologies aimed to improve the microbial safety of the food supply. Commercial SBIR cameras built with distinct features (e.g., lenses and infrared detectors), temperature ranges, and frame rates are readily available at different costs. Even more, they have different accuracy and thermal resolution levels. This creates an opportunity to select a good SBIR camera for a specific application. Historically, professional-grade IR cameras have required the use of specialized postprocessing software to analyze temperature profiles of objects or scenes, which may be time-consuming. However, modern SBIR cameras can operate with smartphone image-processing applications that allow the easy import, editing, and analysis of thermal images [18].

Previous studies have compared temperature measurements by traditional temperature monitoring tools and TI techniques. For instance, Badia-Melis, Emond, Ruiz-García, Garcia-Hierro, and Robla Villalba [19] conducted a comparative study between temperatures measured traditionally by temperature probes and the temperatures measured by high-cost handheld thermal cameras. Although IR cameras are widely used in several industrial applications, they may be too expensive for the fresh produce industry. In contrast, the recent availability of SBIR cameras in the market has opened opportunities for the fresh produce industry to adopt these cost-effective technologies. Nevertheless, the effectiveness of using these newly developed technologies as temperature monitoring tools in the fresh produce industry has not been reported. Therefore, our team hypothesized that SBIR cameras could be practical and cost-effective tools for monitoring fresh produce’s cooling and storage temperatures. The objectives of this study were (1) to evaluate the performance of different SBIR cameras under different temperature conditions and (2) to assess simulated immersion hydrocooling and cold storage conditions of fresh produce using SBIR cameras.

## 2. Materials and Methods

### 2.1. Materials

SBIR cameras and thermal imagers: Four SBIR cameras were evaluated, including the FLIR One Pro (Teledyne FLIR, Arlington, VA, USA) (for Android and iOS devices) and Seek Thermal Compact Pro (Seek Thermal, Santa Barbara, CA, USA) (for Android and iOS devices). Moreover, two handheld thermal imagers were acquired, including the FLIR CX5 (Teledyne FLIR, Arlington, VA, USA) and the Seek Thermal Shot Pro (Seek Thermal, Santa Barbara, CA, USA). The preliminary description of SBIR cameras and handheld thermal imagers are shown in Table 1.

Fresh produce: Refrigerated iceberg, large and/or small romaine lettuce heads as well as fresh cantaloupes were obtained from a local supermarket (Griffin, GA, USA). Upon receipt, the lettuces and cantaloupes were stored in a cold room at 4 °C until use.

### 2.2. Evaluation of SBIR Cameras and Thermal Imagers

The performance of thermal cameras was evaluated at different environmental temperatures. First, lettuce heads and cantaloupes were placed in cardboard boxes. Then, thermal images of the fresh produce were acquired with SBIR cameras and handheld thermal imagers at three different temperatures (~35 °C, ~20 °C, and ~4 °C) to simulate outdoor, packinghouse, and refrigerated environments, respectively. The emissivity value (ε) was set at 0.95. A previously calibrated food traceable thermometer (Traceable, Webster, TX, USA) was also used for the surface temperature validation.

### 2.3. Simulated Inmersion Hydrocooling

#### 2.3.1. Simulated Hydrocooling of Lettuces

Refrigerated lettuces were incubated at 42 °C for 20 h in an environmental chamber (Fisher Scientific Inc., Dubuque, IA, USA) to increase the temperatures of the fresh produce and mimic field temperatures after harvesting. Then, eight lettuce heads were placed in a stainless-steel tank containing 15 L of chilled water (~0.4 °C) for up to 90 min to simulate immersion hydrocooling.

#### 2.3.2. Simulated Hydrocooling of Cantaloupes

Refrigerated cantaloupes were placed in an environmental chamber at 42 °C for 20 h. Then, the warm cantaloupes were immersed in 15 L of chilled water (~4 °C) for up to 2 h to simulate immersion hydrocooling.

#### 2.3.3. Temperature Measurements

**Figure 1:**
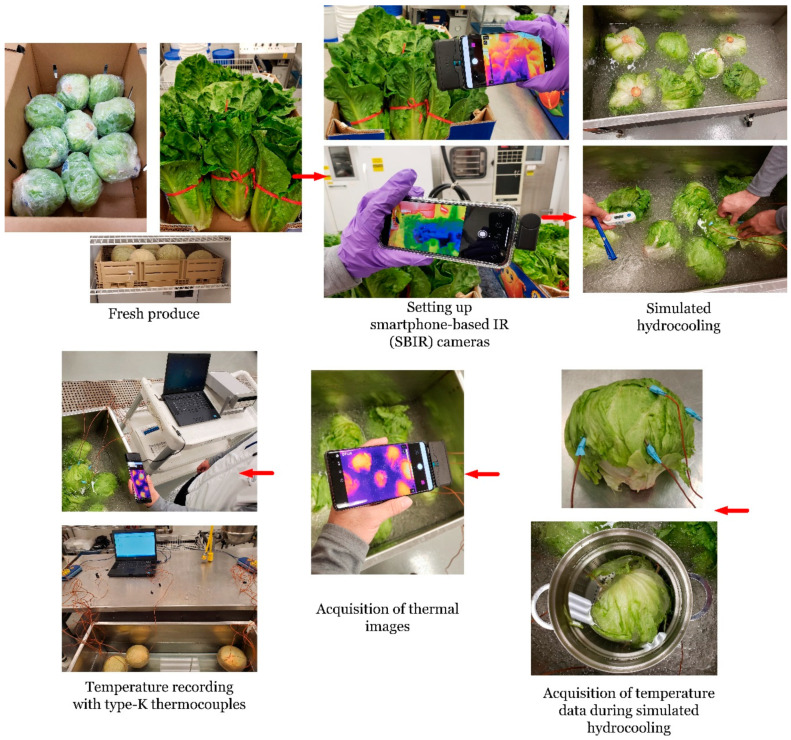
Simulated immersion hydrocooling of romaine lettuce, iceberg lettuce, and cantaloupes.

Thermal images of the fresh produce were taken before, during, and after simulated immersion hydrocooling using SBIR cameras and handheld infrared cameras. The acquired thermal images reported temperature profiles in a degree Celsius scale. The surface and internal temperatures of the fresh produce was continuously recorded in degree Celsius at 10 s intervals using type-K thermocouples (Model TC-08, Omega, Norwalk, CT, USA) connected to a data acquisition system (Model PT-104A, Omega, Norwalk, CT, USA). The experimental setup of the simulated immersion hydrocooling experiments is shown in Figure 1.

### 2.4. Storage Temperatures of Fresh Produce in the Walk-in Cooler

#### 2.4.1. Walk-in Cooler Storage

After simulated immersion hydrocooling, cold lettuces and cantaloupes were placed in cardboard boxes which were immediately stored at different heights in a walk-in cooler set at 4 ± 2 °C (Enviro-line rooms, Norlake Inc., Hudson, WI, USA) for up to three days.

#### 2.4.2. Temperature Measurements

**Figure 2:**
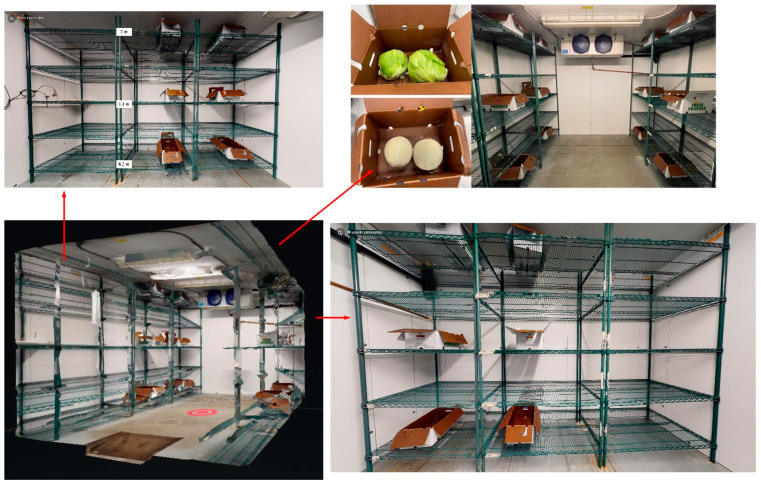
Walk-in cooler storage of fresh iceberg lettuce and cantaloupes at different height positions (upper, middle, and lower). Three-dimensional model of walk-in cooler can be accessed at: https://my.matterport.com/show/?m=966gToxUj5D (accessed on 21 October 2022).

After evaluating the six thermal cameras in terms of accuracy, thermal image quality, and processing time as described in Section 2.2 and Section 2.3, the preferred SBIR camera was selected to monitor the surface temperature of the fresh produce during the simulated walk-in cooler storage. A food traceable thermometer was also used for the surface temperature validation. Additionally, temperature data loggers (Digi-sense, model 20250-3, Cole-Parmer, Vernon, IL, USA) were used to record the air temperature at different height positions in the walk-in cooler. The experimental setup of the walk-in cooler storage is shown in Figure 2.

#### 2.4.3. Three-Dimensional (3D) Model of Walk-in Cooler

Virtual models of a walk-in cooler were created using a state-of-the-art 360-camera (Matterport Pro2, Matterport Inc., Sunnyvale, CA, USA) and cloud-based software Matterport. The resultant virtual model was manually assembled and assessed based on its precision, accuracy, resolution, and easiness of manipulation. The collected footage was completed in a single take and the resultant virtual model could be accessed online through a computer, smartphone, or with a virtual reality (VR) headset.

### 2.5. Statistical Analysis

All experiments and analyses were carried out in triplicate determinations. Means and standard deviations of experimental results were reported, and the data were then analyzed using statistical software SAS (SAS OnDemand for Academics, SAS Institute, Cary, NC, USA). The significance of the observed differences among the means of experimental results was evaluated by an analysis of variance (ANOVA) and a post hoc Tukey’s studentized range test (α = 0.05).

## 3. Results

### 3.1. Evaluation of Thermal Cameras

**Figure 3:**
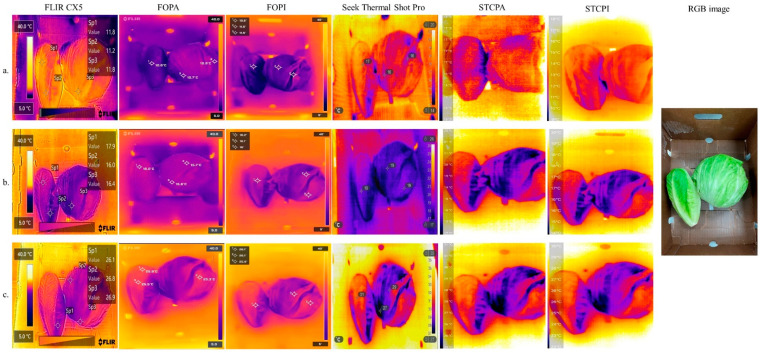
Thermal images of lettuce heads taken from six thermal cameras operated at (**a**) 4 °C, (**b**) 20 °C, and (**c**) 35 °C. FOPA = FLIR one Pro connected to an Android smartphone; FOPI = FLIR one Pro connected to an iOS smartphone; STCPA = Seek Thermal Compact Pro attached to an Android smartphone; STCPI = Seek Thermal Compact Pro attached to an iOS smartphone; RGB = red, green, and blue.

Thermal images of fresh romaine and iceberg lettuces taken from six different thermal cameras are shown in Figure 3. After keeping the thermal cameras and fresh lettuces at 4, 20, and 35 °C for 30 min (to allow temperature equilibrium), the surface temperature profiles of the lettuces taken by FLIR series thermal cameras at 4 °C were drastically different from those taken by Seek thermal cameras (Figure 3). The temperature range of a randomly selected area from the lettuces’ surface taken by FLIR CX5 was 9.8 to 13.5 °C. Meanwhile, the mean surface temperature of three randomly selected spots on the lettuces taken by FOPA (FLIR one Pro connected to an Android smartphone) and FOPI (FLIR one Pro connected to an iOS smartphone) was 10.73 ± 0.15 °C and 11.20 ± 0.52 °C, respectively. Temperature readings monitored by FLIR series infrared cameras were validated with a previously calibrated thermometer (Table 2). However, temperature maps obtained from Seek series thermal cameras were less accurate since the obtained thermal data were significantly (*p* < 0.05) higher (from 3 °C to 5 °C) than those obtained by FLIR cameras. Similar results were obtained when the temperature readings were taken at 20 and 35 °C.

**Figure 4:**
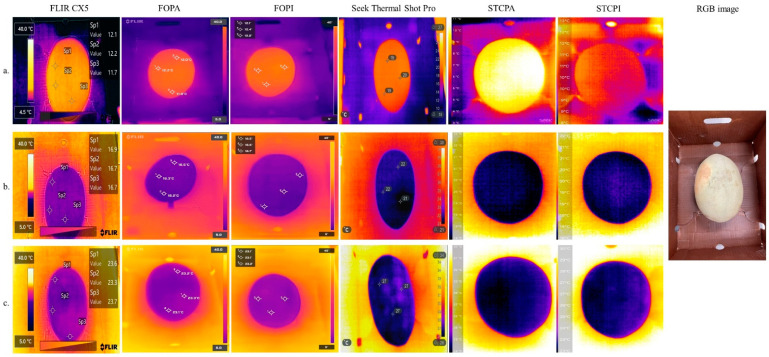
Thermal images of cantaloupes taken from six thermal cameras operated at (**a**) 4 °C, (**b**) 20 °C, and (**c**) 35 °C. FOPA = FLIR one Pro connected to an Android smartphone; FOPI = FLIR one Pro connected to an iOS smartphone; STCPA = Seek Thermal Compact Pro attached to an Android smartphone; STCPI = Seek Thermal Compact Pro attached to an iOS smartphone; RGB = red, green, and blue.

Similarly, thermal images of the fresh cantaloupe taken from six different thermal cameras are shown in Figure 4. Although, temperature profiles captured by all SBIR cameras were within ±2 °C of the temperature readings obtained with a calibrated thermometer, the thermal profiles captured by FLIR series cameras under three differently conditions were still more precise than those captured by Seek series cameras. Additionally, the thermal maps of the cantaloupe had stronger contrasts against the background (i.e., everything else) than that of the lettuces, indicating that lettuces were more susceptible to the surrounding environment.

### 3.2. Simulated Immersion Hydrocooling

**Figure 5:**
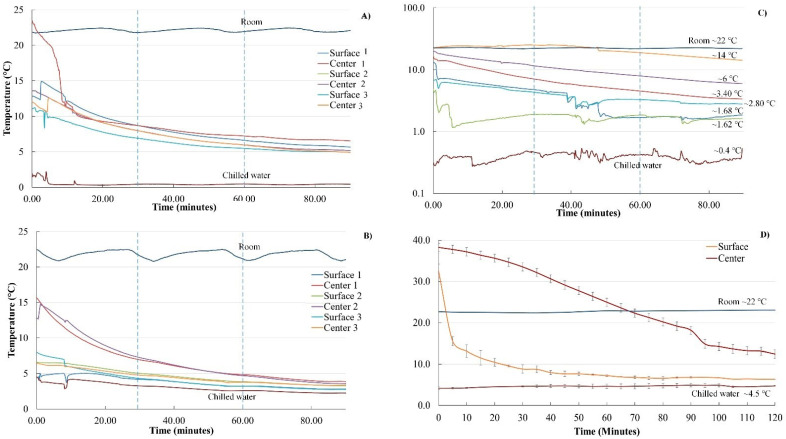
Surface and internal temperatures (°C) of large romaine lettuce heads (**A**), small romaine lettuce heads (**B**), iceberg lettuce heads (**C**), and cantaloupes (**D**) during simulated immersion hydrocooling.

**Figure 6:**
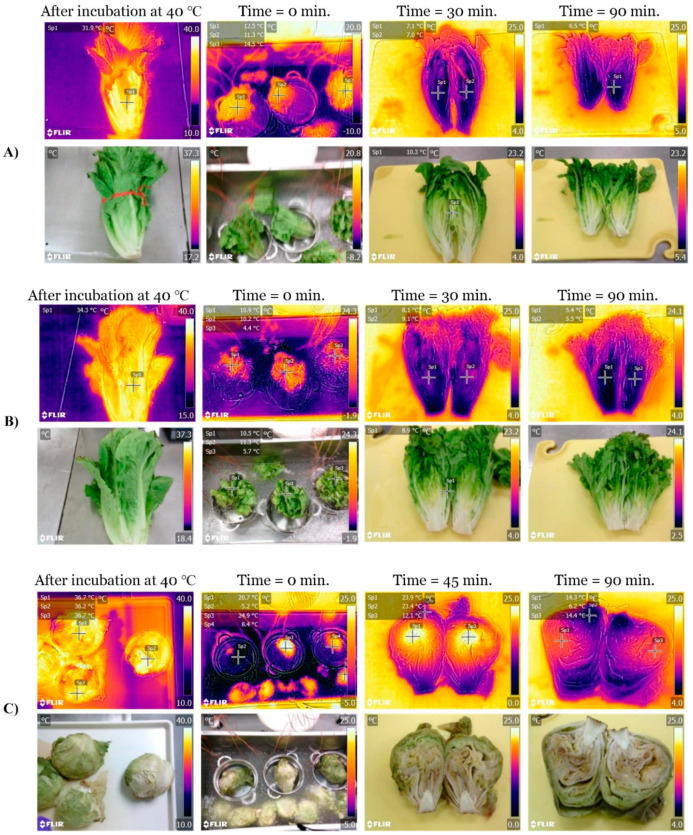
Thermal images (taken at room temperature of ~22 °C, relative humidity ~55%) of small romaine lettuces (**A**), large romaine lettuces (**B**), and iceberg lettuces (**C**) stored at 40 °C for 20 h, and during simulated hydrocooling after 0, 30, and 90 min. Thermal images were taken with a FLIR CX5 camera.

The surface and internal temperatures (obtained with thermocouples) of the lettuce heads during simulated hydrocooling are shown in Figure 5. The simulated immersion hydrocooling of the lettuces was conducted for 90 min. Room temperature was kept at ~22 °C; meanwhile, chilled water temperature was kept constant (~0.4 °C). As expected, after incubation at ~42 °C for 20 h, the surface and internal temperatures of the lettuce heads were >25 °C. As shown in Figure 5A–C, the cooling curves of the lettuces showed a moderate temperature drop at the beginning of the simulated immersion hydrocooling experiment, which corresponded to heat lost (sensible heat) from the product to the chilled water. After 1 h of hydrocooling, the surface temperature of the small romaine lettuces was approaching 5 °C (Figure 5A,B). Not surprisingly, the internal temperature was always higher than the surface temperature of the lettuces; this effect was more evident in iceberg lettuces (Figure 5C). Interestingly, the internal temperature of some large lettuce heads never reached 5 °C even after 90 min of simulated immersion hydrocooling (Figure 5C). These results were confirmed with thermal images taken with FLIR series thermal cameras (Figure 6). Lettuce heads were taken every 30 min and cut in half to take thermal images, which revealed higher internal temperatures than the surface temperatures during the simulated immersion hydrocooling procedure as reported by previous work [20].

**Figure 7:**
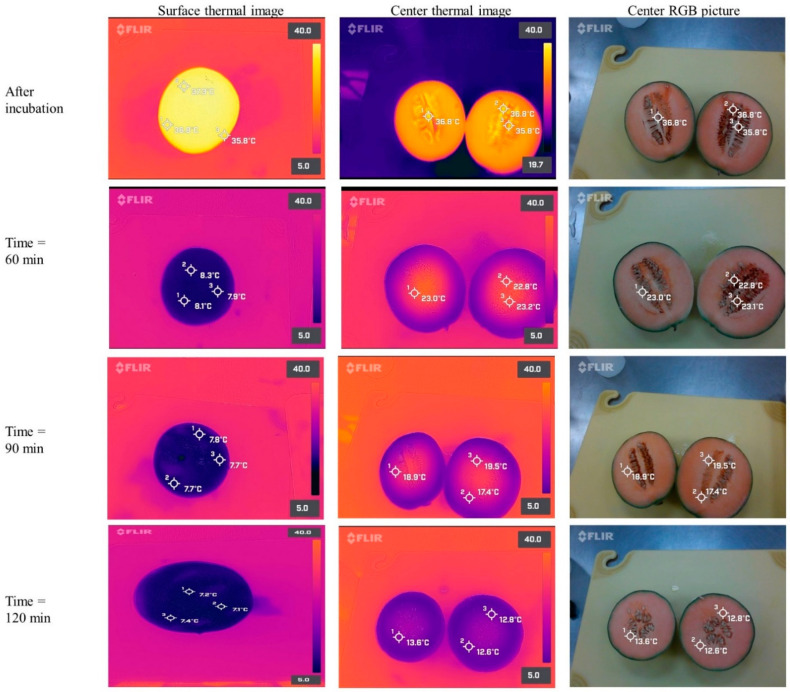
Thermal images (taken at room temperature of ~22 °C, relative humidity ~55%) of cantaloupes incubated at 42 °C for 20 h, and during simulated immersion hydrocooling after 0, 60, 90, and 120 min. Thermal images were taken with a FLIR One Pro camera connected to an Android smartphone.

Similarly, the cooling curves of the warm cantaloupes revealed that the surface temperatures rapidly decreased during the first 30 min of simulated immersion hydrocooling (Figure 5D). Furthermore, the internal temperatures were higher than the surface temperatures of the cantaloupes during the two hours of simulated immersion hydrocooling. It is essential to highlight that the internal temperatures of the cantaloupes were ~18 °C after 90 min and did not reach 10 °C even after two hours of simulated hydrocooling. Thermal images obtained with FLIR series infrared cameras confirmed those results (Figure 7).

### 3.3. Storage Temperature of Fresh Produce Inside the Walk-in Cooler

**Figure 8:**
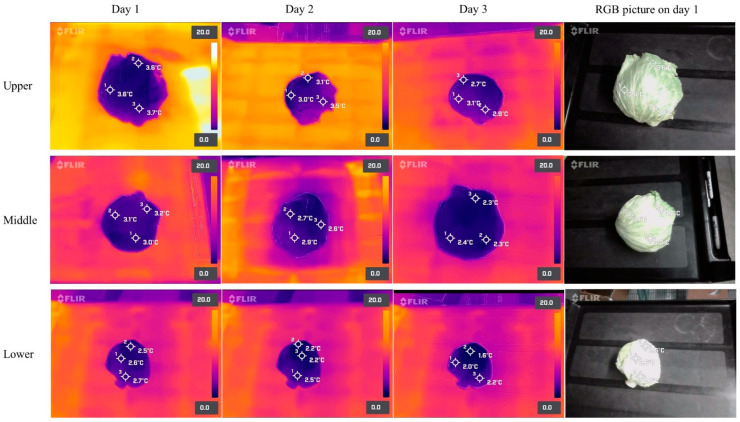
Thermal images of iceberg lettuces at different height positions (upper, middle, and lower) during 3 days of walk-in cooler storage. Thermal images were taken with a FLIR One Pro camera connected to an Android smartphone.

**Figure 9:**
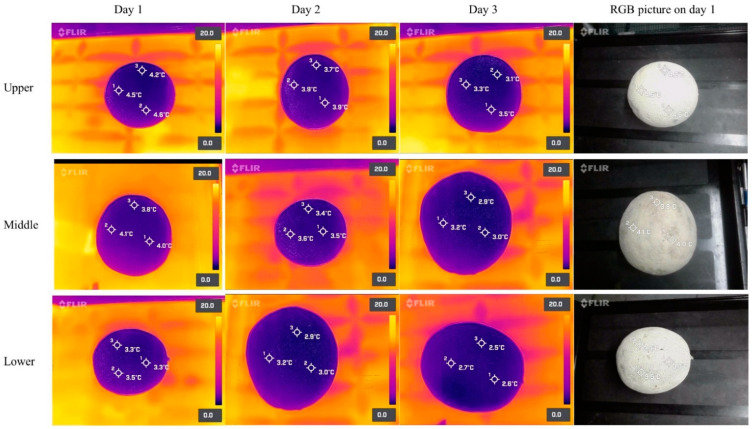
Thermal images of cantaloupes at different heights (upper, middle, and lower) during three days of walk-in cooler storage. Thermal images were taken with a FLIR One Pro camera connected to an Android smartphone.

After 24 h of refrigerated storage, the mean surface temperatures of the lettuce heads stored at the higher (2 m from the ground), middle (1.1 m from the ground), and lower (0.2 m from the ground) shelf positions were 3.6 ± 0.27 °C, 3.12 ± 0.13 °C, and 2.36 ± 0.1 °C, respectively (Table 3). Then, after three days of storage, the surface temperature of the lettuce heads at higher, middle, and lower shelf positions dropped to 2.78 ± 0.21 °C, 2.24 ± 0.16 °C, and 1.71 ± 0.06 °C, respectively. Interestingly, there was a significant (*p* < 0.05) difference in surface temperature between the lettuces stored at different height positions after three days of refrigerated storage. Thermal images taken with FOPA cameras confirmed those results (Figure 8). Similarly, the surface temperature of cantaloupes during three days of walk-in cooler storage is shown in Table 3. After one day of refrigerated storage, the surface temperature of the cantaloupes placed at higher, middle, and lower shelf positions was 4.32 ± 0.1 °C, 3.78 ± 0.19 °C, and 3.31 ± 0.03 °C, respectively. The surface temperature of the cantaloupes dropped ~0.3–0.4 °C every 24 h, started to plateau after three days, and was significantly (*p* < 0.05) higher for those stored in higher positions compared to lower shelf positions. After three days of refrigerated storage, the surface temperature of the cantaloupes was 3.41 ± 0.09 °C, 3.09 ± 0.06 °C, and 2.73 ± 0.09 °C at the higher, middle, and lower shelf positions, respectively. Thermal images obtained from FOPA cameras confirmed those findings (Figure 9).

## 4. Discussion

### 4.1. Evaluation of Thermal Cameras under Three Different Temperature Environments

Previous studies have reported emissivity values close to 0.95 for fruits and vegetables [21]. Although the emissivity value can be fairly adjusted in the SBIR cameras and thermal imagers, our team decided to use the emissivity value of 0.95 for practical purposes. To effectively monitor apples’ surface temperature for timely actuation of remedial measures, Wang et al. [18] developed a smartphone-based tool assisted with thermal–RGB cameras (FLIR One Pro, FLIR Systems, Inc., Wilsonville, Oregon, OR, USA) for monitoring apple fruits’ surface temperature. The acquired imagery data were processed on a custom-designed application called “AppSense 1.0” that helped to manage and share the data with end-users. The study suggested that the integrated tool was reliable to estimate surface temperature of four most common apple cultivars grown in the state of Washington, USA.

As shown in Figure 3 and Figure 4, the thermal images of the cantaloupe had stronger contrasts against the background than those of the lettuces. The intense contrast between thermal images of objects and background would be helpful in the segregation of the objects by removing unrelated areas or pixels in the infrared pictures [22]. For instance, Ranjan et al. [12] utilized a K-mean + + clustering-based unsupervised classification algorithm to remove the background from apples. To improve the quality of the image and accuracy, the researchers used a multimodal imaging system that integrated an RGB sensor with a thermal imager (FLIR Lepton 2.5, FLIR Systems, Inc., Wilsonville, OR, USA). The system allowed the identification of minor temperature differences between apples and their background. In this study, the resultant thermal images indicated that additional imaging techniques to achieve segregation from their background were not required. Moreover, as shown in Figure 4, thermal images acquired from FLIR series cameras enabled a better contrast between objects and backgrounds than those acquired from Seek series cameras, especially under cooling conditions (Figure 4a).

Both FLIR and Seek cameras are based on TI technology, but they differ in their unique features such as the design of thermal sensors, image enhancement techniques, field of view (FOV), and algorithms for signal processing [23]. The main factor that affects the quality of the thermal images is focus [24]. Focus can directly affect the quality and accuracy of the obtained thermal images because a blurred focus reduces the clarity of the energy absorbed by the thermal camera, while a sharp focus can precisely measure that energy and assign accurate temperature values to it. Both FLIR and Seek handheld IR cameras have focusable lens. However, Seek SBIR cameras have manual focus systems that increase the depth of field. Meanwhile, FLIR SBIR cameras have an autofocus feature that does not require a sophisticated method to determine the correct focusing distance. FOV is also important for thermal image quality because it determines how broad an image a thermal camera can capture [23]. The FOV of the FLIR CX5, FLIR One Pro, Seek Thermal Shot Pro, and Seek Thermal Compact cameras is 54°, 43°, 57°, and 32°, respectively. A higher FOV indicates the extent of a scene that a camera will see at any given moment. For close-up work, thermal cameras with a FOV of 45 or higher are required, meanwhile, for long-distance applications, thermal cameras with a FOV between 12 and 6° are preferred.

In this study, FLIR SBIR cameras were more effective and user-friendly than Seek SBIR cameras, regardless of the smartphone type. FLIR SBIR cameras were easily manipulated with the integrated App FLIR ONE. Furthermore, the collected thermal images could be effectively processed and shared with smartphones, which makes the SBIR cameras practical tools for temperature measurements in packinghouses of fresh produce. It was noted that FLIR SBIR cameras had a limited battery life (~50 min) when used continuously. Although Seek SBIR cameras offered a higher thermal resolution than FLIR SBIR cameras, and their power source was the smartphone’s battery (a feature that is convenient if the thermal camera is used for extended periods), focused thermal images were difficult to acquire with Seek SBIR and the Seek Thermal App was extremely limited and not user-friendly, especially because it lacked image-editing features.

In contrast, the FLIR One Pro had a lower thermal resolution than the Seek SBIR and Professional grade IR cameras, which resulted in fewer data points in a thermal image. Nonetheless, it was noticed that the thermal resolution of FLIR SBIR cameras was good enough to monitor the surface temperature profiles of fresh produce under three different temperature conditions. Seek SBIR cameras can acquire temperature readings from −40 °C to 330 °C. Meanwhile, FLIR SBIR cameras can measure temperatures from −20 °C to 400 °C. In addition, excellent quality thermal images were taken with both iOS and Android devices; however, higher quality images were taken when SBIR cameras were connected to an Android smartphone.

We noted that the performance of the IR thermal imagers was impressive. They had good thermal resolution and battery life (~4 h). Their construction was robust, and their software was user-friendly easy to manipulate. Both devices offered Wi-Fi connectivity which facilitated data sharing and storage. Nevertheless, the FLIR CX5 model was preferred over the Seek Thermal Shot Pro during the experiments due to a distinctive feature called MSX (multispectral dynamic imaging) that is exclusive to FLIR cameras. MSX adds visible light details to thermal images in real time to achieve a greater clarity without diluting the thermal image or decreasing thermal transparency [25]. Additionally, FLIR CX5 was faster at processing and analyzing heat signatures. Therefore, we selected FLIR series thermal cameras for conducting the rest of the experiments.

### 4.2. Simulation of Immersion Hydrocooling

As shown in Figure 5C, the internal temperature of the large lettuce heads never reached 5 °C even after 90 min of simulated hydrocooling. It has been reported that the cooling rate of fresh produce (heat transfer) may be affected by produce geometry, such as size, shape, heat transfer coefficient, and the surface-to-volume ratio [26]. Additionally, the inadequate cooling time used in this study could also lead to insufficient cooling. Dincer [27] developed a mathematical model to determine the heat transfer coefficients of both spherically and cylindrically shaped food products during hydrocooling at different water temperatures. The results indicated that the developed model incorporating heat transfer coefficient, lag factor, and produce properties could be used for food products with various shapes. França et al. [28] evaluated the cooling rate of butter lettuce during immersion hydrocooling. The initial internal temperature of the lettuces was approximately 20 °C. The results showed that immersion hydrocooling could exponentially reduce the temperature of the lettuce, and the final internal temperature was stabilized at around 8 °C after 5 min. Similarly, Aroucha et al. [29] found that hydrocooling was effective in maintaining the firmness and soluble solids of cantaloupe fruits and minimizing the precooling time in the forced air circulation tunnel. However, the hydrocooling did not extend cantaloupes shelf life. It was concerning that both thermal images and temperature measurement by thermocouples showed that the internal temperature of the produce was always higher than the surface temperature of the produce during simulated immersion hydrocooling (Figure 5, Figure 6 and Figure 7). This suggests that the internal structures of the produce should also be considered as an essential factor for designing precooling systems, and the simulated immersion hydrocooling conditions were not effective at reducing the temperatures of lettuce heads to acceptable levels (~5 °C), which might be a concern if the cold surface lettuces with a warm core are further stored and processed for the fresh-cut market due to inadequate removal of field heat from lettuces, leading to the accelerated metabolic processes and the increased growth of microorganisms [30]. Furthermore, inefficient precooling could cause temperature fluctuations during cold storage and thus increase the required refrigeration capacity of the cold storage room [7].

Although the aim of the study was not to determine the effectiveness of immersion hydrocooling, it was noted that the immersion hydrocooling approach used in the study was not effective enough to cool down the produce, suggesting that immersion hydrocooling may not be suitable for all types of fruits and vegetables. Forced air cooling and vacuum cooling are the other commonly used precooling methods. Forced air cooling forces cold air to quickly flow through bulk produce and palletized produce [3]. The speed and temperature of the cold air are critical factors which affect the effectiveness of forced air cooling. High air velocity and low air temperature can reduce the cooling time, but they also lead to a high energy consumption and result in water loss and chilling injury of the fresh produce [3,31]. Therefore, it is essential to select an appropriate air flow rate and temperature as well as cooling time for various fruits and vegetables. Additionally, numerous studies have reported to combine forced air cooling and controlled atmosphere storage to inhibit produce reaction heat and extend shelf life [32,33]. The principle of vacuum cooling is based on the evaporative heat transfer mechanism. During vacuum cooling, the pressure in the cooling chamber is reduced by pumping out the gas, leading to the decrease of the boiling point of water. The reduced boiling point allows the free water to evaporate quickly from both surface and inside of foods, which results in heat removal and rapid cooling [31]. Commercial vacuum cooling systems are available in the market [34]. However, the major drawbacks of vacuum cooling include uneven temperature distribution, hardness increase, and mass loss of foods due to evaporation, which might affect food quality and lead to economic loss for the food industry [35]. Therefore, the optimization of processing conditions and equipment designs are required to improve product quality. Immersion vacuum cooling, water spraying, the optimization of the final pressure, and pressure drop rate are commonly used to improve the temperature distribution and mass loss of food products [36,37,38].

This study demonstrated that FLIR series thermal cameras were promising alternatives for traditional temperature acquisition devices to obtain fresh produce surface temperature profiles during immersion hydrocooling. Moreover, thermal images may support quicker informed decisions to correct process deviations compared to conventional measurements, which are single-point-based and involve a direct contact with the produce. As we mentioned earlier, higher quality images were taken when SBIR cameras were connected to an Android smartphone which utilized more powerful and advanced techniques such as an “Optical Image Stabilization system” to stabilize the camera, and thus delivering clear and sharp photos. This feature is important because it can minimize image quality loss caused by shaky hands during photo taking. Therefore, we used the FOPA for surface temperature measurement of the fresh produce during the walk-in cooler storage.

### 4.3. Storage Temperature of Fresh Produce in Walk-in Cooler

During the walk-in cooler storage, the surface temperature of the lettuces was lower than that of the cantaloupes, suggesting that the cooling air of the walk-in cooler removed heat at a higher rate from the lettuces due to their high surface area (>1400 cm^2^/100 g) compared to the cantaloupes (~400 cm^2^/100 g) [31,39,40]. It is noted that the air temperature obtained from temperature data loggers placed at different height positions in the walk-in cooler constantly fluctuated, which might be caused by human activity in the walk-in cooler and frequent openings and closings of the door during temperature measurements. Additionally, the active physiological and biochemical activities of lettuces and cantaloupes due to insufficient precooling could also lead to temperature fluctuations [26]. Nevertheless, there were still differences in air temperature among height levels. A high variability and uneven air temperature distributions may be observed inside cold rooms storing fresh produce. This is affected by how the product is stacked, the quantities stored, and the initial temperature of the products [41]. Temperature abuse inside cold rooms may be observed in areas with poor air circulation and high moisture condensation, which may promote rapid product deterioration and the growth of foodborne pathogens if this adverse factor is not adequately controlled, thus representing a food safety concern. However, critical areas (areas where temperature abuse occurs) might be difficult to identify by using conventional tools [42]. Moreover, cold air has a higher density than warm air, and in this study, the cooling air density at the higher, middle, and lower shelf positions was around 1.274 kg/m^3^, 1.279 kg/m^3^, and 1.284 kg/m^3^, respectively, suggesting that the cold air sat at the bottom of the walk-in cooler. Therefore, the application of instruments that can generate temperature readings in real time such as TI techniques for mapping the temperature distribution inside the cold storage room are essential to verify the efficiency of storage conditions [41].

Since the outbreak of the COVID-19 pandemic, people prefer to work remotely. Therefore, we developed a virtual model of the walk-in cooler (Figure 2) (available at: https://my.matterport.com/show/?m=966gToxUj5D, accessed on 21 October 2022), hoping that the virtual tour of the walk-in cooler would help to communicate results more effectively. Moreover, thermal images of fresh produce, obtained by produce handlers, can be sent to managers and/or engineers of packinghouses who can remotely evaluate the cooling and storage conditions of fresh produce in “real time” and at an affordable price. Future efforts might be the integration of thermal images into the virtual model through cross-disciplinary co-collaboration. Gorman, Hoermann, Lindeman, and Shahri [43] developed and evaluated a VR classroom using a VR technique combined with 360-degree images and videos of food safety messages for secondary school students in New Zealand during the COVID-19 lockdown and pandemic. The study demonstrated that VR classrooms could be used to add depth to students’ learning in food safety, since students were highly motivated and engaged in the developed virtual environment.

## 5. Conclusions

Several SBIR and two IR thermal imagers were evaluated under different conditions. In general, FLIR SBIR cameras were better instruments than Seek SBIR cameras (quality of images, ease of postprocessing, etc.) to monitor the temperatures of fresh produce at different environmental temperatures, during simulated hydrocooling and cold storage. Moreover, thermal images can be easily read and used to make quicker decisions to correct process deviations compared to conventional temperature measurements. The simulated immersion hydrocooling approach used in this study was not effective enough to decrease the internal temperature of the fresh produce to a safe level. Other precooling methods such as forced-air cooling and spray hydrocooling with a shower system are recommended to cool fruits and vegetables more rapidly due to their higher air/water flow rate to increase the heat transfer coefficient. During cold storage, the FLIR camaras could still obtain precise and faster results compared to regular temperature-acquisition devices.

The study demonstrated that SBIR cameras could be practical tools to monitor the surface temperature of fresh produce during cooling and cold storage. The fresh produce industry may effectively evaluate their current hydrocooling and/or vacuum cooling as well as storing practices with SBIR cameras. In general, SBIR cameras are a practical, easy-to-use, and cost-effective temperature-monitoring alternative that allows a quick response to unexpected process deviations that may compromise the temperature of fresh produce during postharvest handling and storage.

## Figures and Tables

**Table 1:** Description of SBIR cameras and thermal imagers evaluated in the study.

Model	Platform	IR Resolution	Spectrum Range	Power Source	Operating Time	Object Temperature Range	Accuracy
FLIR one Pro	Android	160 × 120	8–14 μm	Internal battery	1 h	−20 °C~400 °C	±3 °C or ±5%
	iOS						
Seek Thermal Compact Pro	Android	320 × 240	7.5–14 μm	Smartphone	Depends on the smartphone’s battery life	−40 °C~330 °C	
	iOS						±3 °C or ±5%
FLIR CX 5 *	N/A	160 × 120	8–14 μm	Battery	4 h	−20 °C~400 °C	±3 °C or ±3%
Seek Thermal Shot Pro *	N/A	320 × 240	7.5–14 μm	Battery	4 h	−40 °C~330 °C	±3 °C or ±5%

* Does not require the use of a smartphone.

**Table 2:** Mean surface temperature of fresh produce taken with a calibrated thermometer in different environments ^†^.

Produce	Refrigerated	Packinghouse	Outdoor
Lettuce	10.66 ± 0.61 °C ^a^	15.98 ± 0.31 °C ^b^	25.92 ± 0.43 °C ^c^
Cantaloupe	12.12 ± 0.26 °C ^a^	16.68 ± 0.32 °C ^b^	23.22 ± 0.36 °C ^c^

^†^ Values are the mean ± standard deviation of triplicate determinations. ^a–c^ Means with the same letter in the same row are not significantly different (*p* < 0.05).

**Table 3:** Surface temperature of fresh produce take by thermometer during 3 days of walk-in cooler storage ^†^.

Storage Time	Produce	Shelf Position		
		Higher *	Middle *	Lower *
24 h	Lettuce	3.60 ± 0.27 °C ^a^	3.12 ± 0.13 °C ^b^	2.36 ± 0.10 °C ^c^
	Cantaloupe	4.32 ± 0.10 °C ^a^	3.78 ± 0.19 °C ^b^	3.31 ± 0.03 °C ^c^
48 h	Lettuce	3.11 ± 0.24 °C ^a^	2.71 ± 0.20 °C ^b^	2.00 ± 0.15 °C ^c^
	Cantaloupe	3.89 ± 0.11 °C ^a^	3.44 ± 0.15 °C ^b^	2.95 ± 0.10 °C ^c^
72 h	Lettuce	2.78 ± 0.21 °C ^a^	2.24 ± 0.16 °C ^b^	1.71 ± 0.06 °C ^c^
	Cantaloupe	3.41 ± 0.09 °C ^a^	3.09 ± 0.06 °C ^b^	2.73 ± 0.09 °C ^c^

^†^ Values are the mean ± standard deviation of triplicate determinations. ^a–c^ Means with the same letter in the same line are not significantly different (*p* < 0.05). * Higher, middle, and lower shelf positions were 2, 1.1, and 0.2 m above the ground, respectively.

## Data Availability

Data is contained within the article.
